# Who Gives Birth (First) in Female Same‐Sex Couples in Sweden?

**DOI:** 10.1111/jomf.12727

**Published:** 2020-09-12

**Authors:** Katarina Boye, Marie Evertsson

**Affiliations:** ^1^ Stockholm University Sweden

**Keywords:** children, family formation, fertility, longitudinal research, motherhood, same‐sex marriage

## Abstract

**Objective:**

The aim of the study was to analyze factors predicting (a) the transition to parenthood for female same‐sex couples in Sweden and (b) which partner is the birth mother for the first and (any) second child.

**Background:**

Longitudinal studies in which couples become parents are rare for same‐sex couples in any context, even though these families are increasing. Childbearing in lesbian couples is an interesting case for testing theories linked to family utility maximization and household bargaining, as these couples can often choose who will carry a child.

**Method:**

Discrete‐time event history and linear probability models are estimated on Swedish population register data (1995–2016) to analyze couples' transitions to first and second birth and the choice of birth mother.

**Results:**

The higher the household income and partners' educational levels, the more likely couples are to become parents. However, within‐couple income gaps are small, and income and education are unrelated to the choice of first‐birth mother. Couples are more likely to have a second child and to switch birth mothers if both are highly educated or the first social mother is highly educated.

**Conclusion:**

Factors predicting which couples become parents are similar in same‐sex and different‐sex couples. In same‐sex couples, short‐term within‐couple specialization is of little relevance for who becomes the birth mother. Analyses of the transition to a second birth suggest that long‐term planning matters for who becomes the first‐ and second‐birth mother.

## Introduction

In recent decades, families have emerged who previously either faced difficulties forming and/or were not legally recognized. Among them are female same‐sex parent families. Same‐sex parenthood was legally recognized in Sweden in 2003, but even before then, registered partner couples and parents could be identified in population registers. Far from all parents register as partners—and not all couples want to become parents—but research indicates that registered partnership (1995–2008) and marriage (from 2009) has been increasingly common among couples who become parents (Aldén et al., 2015; Kolk & Andersson, 2020). However, little is known about factors predicting the transition to parenthood in lesbian couples. For example, is income important for the transition to first birth in Sweden, where medically assisted insemination or in vitro fertilization (IVF) is part of the national healthcare coverage, costs are low, and treatments have been available to lesbian couples since 2005? What about a potential second pregnancy and birth? The two‐child norm is strong in Sweden (Andersson, 1999), but only medical treatment to have a first child is subsidized, and the parents must carry any costs for medically assisted insemination or IVF for a second child themselves. In this article, the link between partners' age, education, and income and the transitions to first and second birth are estimated for registered partner and married lesbian couples in the period from 1995 to 2016.

Once couples have decided to (try to) become parents, the decision regarding *who will be the birth mother* for the first and potential second child is open to negotiation, unless one of the partners is not in good health or is at the end of her fertile age. Socioeconomic factors and income are pivotal in theories on specialization and the gendered transition to parenthood (e.g., Becker, 1985, 1991). In different‐sex couples, socioeconomic differences are closely interlinked with sex and gender. This makes it difficult to disentangle mechanisms related to, for example, income differences from mechanisms related to gendered behavior and physiological factors such as breastfeeding. Same‐sex couples are a unique test case with regard to assessing sociological and economic theories of the family (Goldberg & Perry‐Jenkins, 2007). Will couples in which sex and earnings are unrelated aim to maximize (short‐term) family utility by letting the person earning the least be the birth mother (cf. Becker, 1985, 1991), or are other factors important for who becomes the birth mother? Lesbian couples may (in theory) take turns giving birth if they desire more than one child. The ways in which resources in terms of income and education play a role in who becomes the first and (if any) second birth mother can provide insights on within‐couple bargaining and reasoning in a supposedly gender‐neutral setting.

To date, we know of no research on the association between socioeconomic factors and the transition to joint parenthood in lesbian couples. In addition, earlier research on within‐couple specialization is predominantly cross‐sectional, retrospective, and based on either surveys or in‐depth interviews with a small group of individuals. Sociodemographic characteristics may be misrepresented in these data due to systematic interview participation and nonresponse (see Gabb, 2004; Henehan et al., 2007).

Sweden was one of the first countries to legally recognize same‐sex partnerships and parenthood, and it is one of the few countries with detailed population register data, enabling large‐scale, longitudinal studies of important life course events and processes in couples' linked lives. Covering the period from 1995 to 2016, this study analyzes factors predicting (a) the probability that female, married, or registered partner same‐sex couples become parents by giving birth to a child and (b) who will be the birth mother in these couples, that is, the person who carries the first and (any) second child.

In the following, we first discuss theories and earlier research on which couples become parents (between‐couple factors) and second, the choice of birth mother in these couples (within‐couple factors). Thereafter, the theoretical background is summarized, and hypotheses are formulated before we turn to a description of the data and the empirical models. The results are presented, and conclusions are discussed in the two final sections.

## Background

### The Motivation for Couples to Become Parents and the Role of Socioeconomic Factors

Focusing on nonfinancial reasons for having children, same‐sex couples and different‐sex couples tend to state similar reasons for wanting to become parents, such as expecting increased happiness, well‐being, and life fulfillment (e.g., Bos et al., 2003; Kleinert et al., 2015). Nevertheless, research indicates that lesbian and gay individuals tend to desire parenthood somewhat less than heterosexual individuals do, partly due to a fear of prejudiced behavior toward themselves or their child (Baiocco & Laghi, 2013; Hayman et al., 2015; Herrmann‐Green & Gehring, 2007; Jennings et al., 2014). Research also suggests that same‐sex couples face more stressors than different‐sex couples while transitioning to and practicing parenthood due to their double minority status: they are parents in a heteronormative context and potentially a minority in the lesbian, gay, bisexual, and transgender (LGBT) community (for an overview, see Cao et al., 2016).

With regard to socioeconomic predictors of couples' transition to parenthood, evidence suggests that income volatility and low levels of household wealth can reduce fertility in different‐sex couples (e.g., Bernardi et al., 2008; Mansour, 2018; Modena et al., 2014). Still, the association may vary by educational level. Kreyenfeld (2016) found that employment uncertainty leads to postponement of birth plans among higher educated women in Germany, whereas employment uncertainty can accelerate childbearing plans among lower educated women. This may be linked to opportunity costs, that is, mothers' foregone earnings during withdrawals from the labor market and the resulting wage penalties, which tend to be higher among the higher educated (e.g., Joshi, 1998; Lappegård & Rønsen, 2005). Still, opportunity costs mainly seem to affect the timing of fertility, at least in the Nordic countries, characterized by a fertility regime with small educational differences in completed fertility (Andersson et al., 2009). Generous parental leave insurances in terms of length as well as reimbursement levels, together with affordable, high‐quality, public childcare reduce the opportunity costs of childbearing. Even though differences still are small, in recent years, childlessness has been the highest among the least educated (Jalovaara et al., 2019). This leads to the expectation of a positive link between education and having first and second birth also for same‐sex couples.

Studies have found a positive relationship between earnings and fertility for women as well as men in different‐sex couples (Andersson, 2000; Hart, 2015). This may be due to the financial investments needed to produce what Becker referred to as “higher quality” children in modern societies (Becker, 1960, p. 211). In other words, educational toys (or cell phones and computers), extracurricular activities, education, and so on are costly, and couples need to have reached a certain income level and/or have secure jobs to afford having children. In a US study, lesbian informants described how having a high paying and middle‐class job provided financial resources and job flexibility that facilitated parenthood (Mezey, 2008). With the exception of Mezey (2008), the studies referred to above focus on different‐sex couples, and as far as we know, there are no large‐scale studies of the link between income and fertility for same‐sex couples.

For lesbian couples, the costs of assisted procreation or adoption add to the regular expenditures of having children. Couples may choose to privately arrange home insemination, but if they do, the social mother must adopt the child to be legally recognized as a parent in Sweden, and in order to adopt, she has to be married to the birth mother. Neither adoption (by the social mother) nor marriage is needed if the couple conceives at a certified clinic, yet the mothers need to cohabit. The major legal differences between marriage and cohabitation have to do with inheritance rights and the way property is divided in case of a divorce (Cohabitees Act, *Sambolag* SFS 2003:376), resulting in more modest legal differences between marriage and cohabitation in Sweden than in many other contexts.

The right to medically assisted procreation within the Swedish healthcare system means that the cost is low for the first child of so‐called involuntarily childless couples. Treatment to have a second child is considerably more expensive (several thousand Euros). Some choose to use clinics abroad even for their first child. Couples may go to Denmark to conceive as a way to reduce the wait, obtain more detailed information about the donor, or be able to choose an anonymous donor (the latter is not an option in Sweden). Couples who use clinics abroad pay for the full treatment, making this solution more expensive than using a Swedish clinic. Worth keeping in mind is that becoming parents and having a child is a well‐thought‐out, planned choice for lesbian couples. No couples become pregnant by accident. Although a comparison to different‐sex couples is not the focus of this study, this most likely contributes to a stronger link between income and parenthood in same‐sex compared with different‐sex couples. Earlier research shows that the birth mother in a same‐sex couple on average has a higher income than the mother in a different‐sex couple (Andresen & Nix, 2019; Evertsson & Boye, 2018; Moberg, 2016). Same‐sex couples are also slightly older than different‐sex couples when they transition to parenthood.

### The Role of Socioeconomic Factors in the Choice of Birth Mother—A Theoretically Informed Perspective

In some female couples, the experience of pregnancy and biological motherhood is more important to one of the partners than to the other (see, e.g., Goldberg, 2006; Hayman et al., 2015). If so, the choice of birth mother may be more or less self‐evident, as long as no medical obstacles (or age) prevent it. In other cases, the choice of birth mother can be seen as a negotiation where the partner with the largest resources in terms of income, education, or labor market position may use this advantage to influence the outcome of the negotiations (cf., Blood & Wolfe, 1960; Lundberg & Pollak, 1996). The desired outcome for each partner in a female same‐sex couple aiming to become parents can be (a) carrying and giving birth to the child or (b) not having to carry and give birth to the child. The theoretical implications suggest that the partner with the largest relative resources can draw on these resources (directly or indirectly) in couple negotiations to arrive at her preferred choice.

Alongside the relative resource or bargaining approach, the specialization theory of Becker (1985, 1991) is commonly used to explain divisions of work and care in families. This theory suggests that couples' wish to maximize household utility explains the gendered division of work. By specializing according to relative advantages in labor market work compared to housework, care, and so on, households maximize their efficiency and thereby their joint income and the utility of household production (of, e.g., childcare). Although Becker expected fewer benefits from specialization in same‐sex couples due to similarities in the partners' human capital, he also expected that even small differences in relative advantages would have long‐term effects on the division of paid and unpaid work. Following the Beckerian argument, family utility is maximized when the partner with the lesser labor market‐related resources in a female same‐sex couple specializes in home production, including the biological investment of carrying and giving birth to a child.

An alternative approach to the specialization perspective is to assume that the partners apply a long‐term perspective when considering who will be the birth mother of the child. If they do, it may be perceived as more financially rational to let the more resourceful partner carry the child (Mezey, 2008). Couples interviewed in the US studies often chose as the birth mother the partner who could take time off from work with the least financial loss, who had the most flexible work schedule, or the best insurance plan (Chabot & Ames, 2004; cf., Goldberg, 2006). Factors such as these correlate with income and education. Still, the income loss from family leave is considerably smaller in Sweden than in the United States. For those who have been employed at least 240 days preceding the birth, the parental leave insurance provides parents with close to 80% of previous income up to a ceiling for the majority of the leave period (in total 480 days that the parents can share between them). Those not eligible for the income‐related leave receive a lower flat rate benefit (180 SEK or close to US $20 per day in the period 2006–2012; Swedish Social Insurance Agency, 2013).

Some couples are most likely already planning for a second child when preparing for the first. In Sweden, the two‐child norm is strong among different‐sex couples (Andersson, 1999). If same‐sex couples aim for two children, they may strive for long‐term family utility maximization, as they negotiate who will be the first and who will be the second birth mother.

## Theoretical Model and Hypotheses

In the empirical analyses, the transition to parenthood is seen as a two‐step process where the decision to become parents is made before the couple decides who will be the birth mother. Hence, which married (or registered partner) couples become parents is empirically analyzed first. Based on the literature review, the expectation is that *couples are more likely to become parents as their joint income and education increase* (Hypothesis 1).

Second, which partner becomes the birth mother is analyzed. Based on theory and earlier research, hypotheses linked to income and education vary. In Table 1, predictions linked to short‐ and long‐term financial reasoning are specified by the choice of the birth mother for the first and (any) second child. According to short‐term, financial specialization theory, *the partner with the lower income and/or education is most likely to be the birth mother* (see Table 1, second column, second row) (Hypothesis 2). This hypothesis is strengthened if couples who have a second child choose the same birth mother for the second as for the first child, holding partners' age constant (Table 1, third column, second row). According to long‐term financial reasoning and the relative resource or bargaining perspective, expectations are that *the more resourceful partner will be the (first) birth mother* (Table 1, second column, third row) (Hypothesis 3). This builds on the assumption that pregnancy and childbirth are less disruptive to women with a strong labor market position. If couples desire two children, they should try to postpone biological parenthood for the least resourceful partner until she has a more secure position or has taken expected career steps. Taking turns to give birth is expected to result in the lowest long‐term financial costs for each of the partners as well as the couple as a whole. Hence, the third hypothesis is strengthened if those who have a second child more often choose the social mother of the first child as the birth mother of the second (Table 1, third column, third row).

**Table 1:** Theoretical Assumptions Regarding Which Partner Is the Birth Mother of a Couple's First and Second Child

Theoretical perspectives	First birth mother	Second birth mother
Short‐term family utility (Becker)	Has the *lowest* income/education of the two	*Same* birth mother as for the first child
Long‐term family utility	Has the *highest* income/education of the two	*Not the same* birth mother as for the first child

## Data and Method

Swedish population register data (the *Total Population Register*, the *Multigenerational Register*, and the *Longitudinal Integration Database for Health Insurance and Labour Market Studies* (LISA)) provided by Statistics Sweden are analyzed. Data cover the entire population and is available annually until 2016. A total of 4,251 female couples who married in the period 1995 to 2016 were identified. A subsample of these (see below for exclusions) were followed annually from the year of marriage until they had a first child and a second child, if any. The first analysis, *the parenthood analysis* (P1), follows couples from registered partnership or marriage and onwards to analyze which couples become parents. The second analysis, *the first birth mother analysis* (M1), analyzes which partner in these couples is the birth mother of the couple's first child. Age is used to discriminate between the two partners, and the younger partner is chosen as the index person. In the third analysis, *the second child and parenthood analysis* (P2), we follow the parental couples from first birth onward and analyze which ones have a second child. Finally, the fourth analysis, *the second birth mother analysis* (M2), estimates the likelihood that both children in two‐child families have the same birth mother.

The descriptive statistics in Table 2 and the multivariate analyses of P1 and M1 exclude 39 couples (0.9% of the initial 4,251 couples) in which it is impossible to discriminate between the partners by age as they were born in the same year and month. A total of 856 couples (20.1% of the initial couples) in which one or both partners had zero work income the year before marriage were also excluded. The reason is that the decision to have a child and the choice of birth mother may be qualitatively different if one or both partners lack income from gainful employment. A total of 15 couples (0.4% of the initial sample) in which one or both partners immigrated to Sweden after the year of marriage were excluded. In addition, 14 couples (0.3% of the initial sample) with missing information on any of the included variables are excluded from the P1 analysis, and five couples are excluded from the M1 and P2 analyses due to missing information. (No couples are excluded from the M2 analysis.) Over time, some women are included as partners in more than one same‐sex couple, but 95% enter a same‐sex marriage only once during the period. Less than 1.5% of the total sample has a child in a second (or higher‐order) marriage.

**Table 2:** Descriptive Statistics for Female Same‐Sex Couples Who Married in the Period 1995–2016

						
	All	Mean/%	*N*	Parental couples	Mean/%	*N*
Annual income 1 year before marriage/partnership^a^	Younger	27,139.67	3,335	First birth mother	27,426.70	1,315
	Older	29,240.60	3,335	First social mother	27,360.01	1,315
	Couple	56,380.27	3,335	Couple	54,786.71	1,315
Annual income 1 year before birth of couple's first child^a^				First birth mother	30,736.66	1,325
				First social mother	30,499.80	1,325
				Couple	61,236.46	1,325
Relative income 1 year before birth of couple's first child						
First birth mother <40% of household income					20.30	269
First birth mother 40%–59% of household inc.					56.91	754
First birth mother ≥60% of household inc.					22.79	302
					100.00	1,325
First birth mother's average relative income 1 year before birth of first child					0.50	2,381
Education first year of marriage/partnership	No high ed.	39.87	1,329	No high education	34.92	462
	Only younger	14.61	487	Only first birth mother	18.37	243
	Only older	20.31	677	Only first social mother	14.89	197
	Both	25.20	840	Both	31.82	421
		100.00	3,333		100.00	1,323
Education the year of birth of couple's first child						
				No high education	32.68	433
				Only first birth mother	18.19	241
				Only first social mother	15.09	200
				Both	34.04	451
					100.00	1,325
Had a child before current marriage/partnership	Younger	13.83	461	First birth mother	8.38	111
	Older	21.33	711	First social mother	10.94	145
Younger partner is first birth mother					54.19	718
Age at birth of couple's first child				First birth mother	33.12	1,325
				First social mother	34.25	1,325
Years between marriage and birth of first child					1.54	1,325
Has a second child^b^					38.26	507
Years between first and second child^c^					2.89	510
Same birth mother for second child^c^					55.49	283

*Note:*^a^Adjusted to 2016 monetary value. In US dollars converted in June 2020. ^b^For the sample in the analysis of birth mother for the first child (M1). ^c^For the sample in the second birth mother analysis (M2).

In summary, the P1 analysis is estimated on a sample of 3,335 couples who contribute 14,445 couple years. The M1 analysis is based on the P1 subsample who had a child: 1,325 couples. The P2 analysis is estimated on 1,336 parental couples who contribute 5,224 couple years. This sample is slightly larger than the sample in the M1 analysis, because couples where the partners were of the exact same age are included in the P2 (but not the M1) analysis. The M2 analysis is run on a sample of 510 two‐child families.

### The First and Second Transition to Parenthood

A discrete‐time event history model is used for the analysis of which couples become parents where the event is the birth of the couple's first (P1) child and second (P2) child. Episodes are censored in 2016 (the last year of observation) or at the time of divorce, emigration, or death of any of the partners. As the dataset consists of couple‐years, *SEs* are clustered at the couple level.

Linear probability models (LPMs) are estimated to analyze which partner is the birth mother in couples who become parents (M1) and have a second child (M2). The analyses are cross‐sectional and estimated in the year of birth of the couple's first and second child, respectively. The M1 outcome is a binary variable indicating that the younger partner (the index person) is the birth mother of the child, and the M2 analysis indicates whether the birth mother of the first child is also the birth mother of the second child.

### Variables

*Marriage cohort* is included in the P1 and P2 analyses. In P1, it is divided into the categories 1995–2002, 2003–2004, 2005–2008, and 2009–2016. The cut‐off points correspond to the first year that same‐sex partners could adopt (2003), get assisted procreation at a Swedish clinic (2005), and marry (2009). In P2, the two first categories are collapsed because of the small number of couples who had a second child these years (20 in 1995–2002 and 37 in 2003–2004). The partners may have had children before they entered the current marriage. Hence, dichotomous variables indicate whether any of the partners have a previous biological child. These variables (*having a child before, younger partner/older partner*) are not included in the P2 and M2 analysis, as very few of the parents who have a second child have children from before (13 of the birth mothers of the first child and 29 of the social mothers of the first child).

*Duration of marriage* is included in the P1 and M1 analyses and acts as the time variable in the discrete‐time event history analysis of first parenthood. It is divided into the categories 0–1, 2–3, and >3 years of marriage. The P2 and M2 analyses instead include *years since first child birth*. It is divided into the categories 0–1, 2, 3, 4, and >4 years in P2, and time varying. In M2, years since first child birth is measured in the year of birth of the second child, and because of small cell sizes, the variable is divided into the categories 0–2 years, 3, and >3 years. *Age* of the younger partner (P1 and M1 analyses) and of the first birth mother (P2 and M2 analyses) in the year of marriage is categorized into 18–30, 31–35, and >35 years of age. The analyses also include the *age difference*; in the P1 and M1 analyses, whether the older partner is at least 4 years older than the younger partner, and in the P2 and M2 analyses, whether the first birth mother is at least 4 years younger or 4 years older than the first social mother.

The P1 and P2 analyses include the logarithm of the sum of the partners' annual gross work incomes (*household income* (*log*)). The logarithmic transformation reduces the likelihood that influential outliers bias the results. Household income is time‐varying and lagged by 1 year in the P1 analysis and measured the year before birth in the P2 analysis. The M1 analysis includes the *younger partner's income* and her *relative income* (her income as a share of the household income) the year before birth. The measure is relevant for estimating short‐term family utility and partners' specialization in paid and unpaid work. For those who have their child early in the year, the income the year before may be influenced by pregnancy‐related absences from work. Therefore, additional analyses (available on request) were estimated with income measured 2 years before birth, with similar results as those presented later. The M2 analysis includes the *income* and *relative income* of the *first birth mother* the year before first birth in order not to let the use of parental leave influence the two measures.

*Education* indicates whether one or both partners have a 3‐year or longer university education. It is time varying in P1 and P2 and measured in the year of birth of the first child in M1 and in the year of birth of the second child in M2. Education is linked to job security and the likelihood of occupying a white‐collar job. Individual measures of education, as well as measures distinguishing between more educational levels, have been tested and gave similar results as those presented here (analyses available on request).

## Results

### Descriptive Statistics

**Figure 1:**
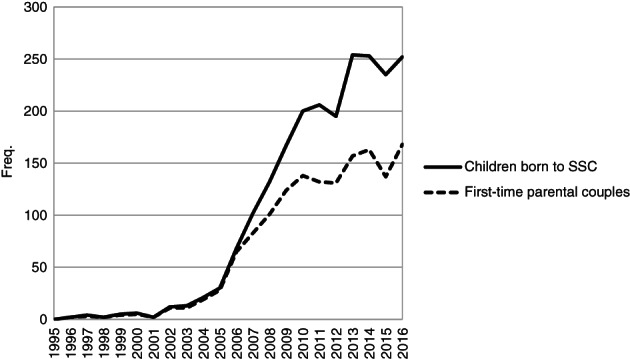
Number of First‐Time Parental Couples and Number of Children Born to Married Female Same‐Sex Couples 1995–2016.

Figure 1 shows the number of first‐time parental couples and the number of children born in these couples in the period from 1995 to 2016. Parenthood among registered partnered female same‐sex couples was uncommon in the 1990s. However, with the granting of adoption rights in 2003 and assisted insemination and IVF in 2005, childbearing within female, married same‐sex couples began to increase (cf. Aldén et al., 2015; Kolk & Andersson, 2020). In 2013–2016, approximately 250 children per year were born to married lesbian couples in Sweden. This makes up approximately 0.23% of all births (250/110,000 average births; Statistics Sweden, 2017). The share of births in lesbian couples would be larger if cohabiting couples were also included. Since 2006, the number of children born to lesbian couples increased substantially more than the number of first‐time parental couples (Figure 1), and it became increasingly more common for couples to have more than one child.

**Figure 2:**
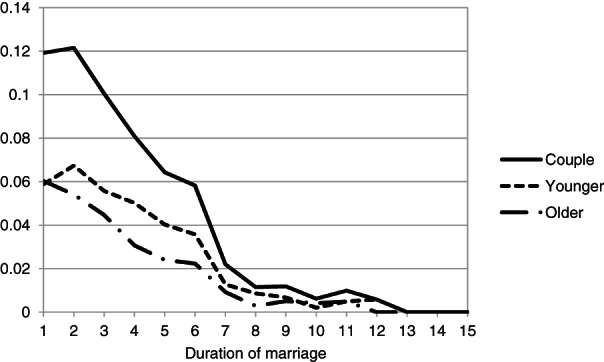
The Share of the Population Under Risk (i.e., Discrete, Annual Hazards) that Has a First Child for the Couple, the Younger Partner, and the Older Partner, by Duration of Marriage in Years.

Most couples had their first child early in the marriage. The average time span between marriage and childbirth was 1.5 years (Table 2). Figure 2 shows that the discrete‐time hazard that a couple had a child decreased after the second year of marriage and was very low after approximately 7 years of marriage. This supports the evidence suggesting that many female same‐sex couples marry as a first step toward parenthood (Aldén et al., 2015; Malmquist, 2015).

**Figure 3:**
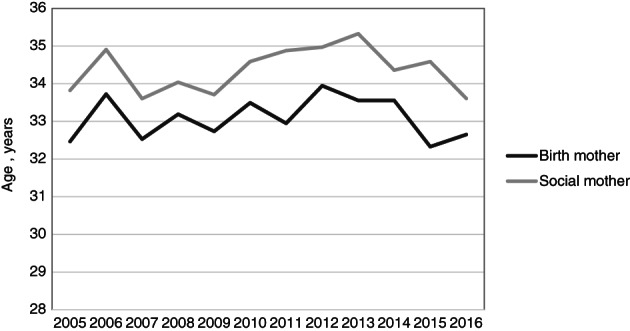
Mean Age at the Birth of the Couple's First Child for the Birth Mother and the Social Mother 2005–2016.

The partners in the couple were equally likely to be the birth mother of the first child if the child was born in the first year of marriage (Figure 2). From the second year on, the younger partner was more likely than the older partner to be the birth mother. It could be that couples try to conceive early in the marriage for physiological reasons if they have chosen the older partner as the birth mother. As time passes, they may be more likely to choose the younger mother. As seen in Figure 3, the birth mother's average age at childbirth was lower than the social mother's age and has been so since 2005 (the graph excludes earlier years due to the low number of first‐time parental couples each year; *n* < 20).

**Figure 4:**
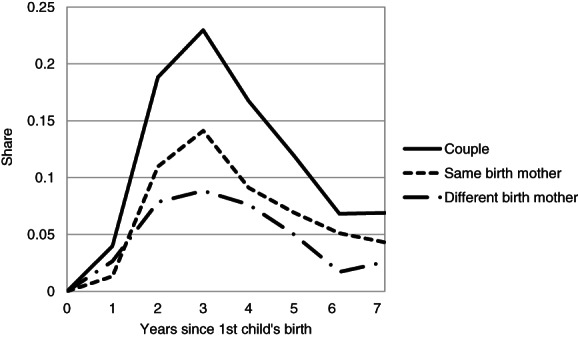
The Share of First‐Time Parents (i.e., Discrete, Annual Hazards) Who Have a Second Child, the Share Where the First and Second Child Have the Same Birth Mother, and the Share Where the First and Second Child Have Different Birth Mothers, by Years Since the Birth of the First Child.

Figure 4 shows the transition to second childbirth among parental couples. This was most common 3 years after the first childbirth and very uncommon 6 years after first childbirth. Beyond the period extending 1 year after the birth of the first child, the first birth mother was slightly more likely to give birth to the couple's second child than the first social mother was. The difference was the largest 3 years after the birth of the first child. During the rest of that period, it was marginally less common to take turns giving birth than it was to let the same partner carry both children. These results are without controls, so one reason for this finding may be that the first birth mother tends to be younger than the first social mother. In supplementary analyses on a smaller sample, those with children from an earlier relationship were excluded. This did not change the pattern, and in these couples, taking turns was slightly less common than having the same birth mother.

Table 2 shows descriptive statistics for the younger and older partners in the sample analyzed in the transition to parenthood analysis (P1) and for the first birth mother and social mother among the parental couples. In the entire sample of couples, the younger partner had a somewhat lower annual income than the older partner had 1 year before marriage. The income difference between the first birth mother (to‐be) and the social mother in the group who became parents was negligible the year before marriage as well as the year before first birth. In more than half of the couples, both partners contributed 40–59% of the household income. Among the rest of the parental couples, the birth mother was about as likely as the social mother to contribute at least 60% of the household income. It was more common for both partners to have higher education in the year of marriage in the (to‐be) parental couples compared to couples who did not have a child in the studied period.

In the parental couples where only one partner was highly educated, the first birth mother was somewhat more likely than the social mother to be the one with higher education.

Childbearing in female‐same sex couples was more common among couples and partners who had not previously had a child, and the first birth mother was slightly less likely than the social mother to have had a previous child. Among the parental couples, 38% had a second child during the studied period. The same partner gave birth to both children in 55% of the couples who had a second child (Table 2). Regarding the likelihood of carrying the second child, the difference between the first birth mother and the social mother was hence small (but statistically significant).

In the following, the results of the event‐history analyses and LPMs are presented. Cell sizes for categorical variables used in these analyses can be found in Appendix. Any associations between control variables and the dependent variable(s) that have been discussed earlier will not be mentioned again if they are observed also in the multivariate analyses later.

### Analyses of the First Childbirth: Parenthood and Birth Mother

The results of the discrete time, event history model of parenthood (P1), and the LPM of first birth mother (M1) are displayed in Table 3. The parenthood analysis shows that couples often became parents early in marriage. To test whether the results are driven by age effects, additional analyses were conducted censoring couples at the year the older partner turned 40 years old. In these analyses (available from the authors), the log odds increased 5–6 years into the marriage, indicating that mainly relatively old couples had their child early in the marriage.

**Table 3:** The Probability that a Female, Same‐Sex Couple Becomes Parents and that the Younger Partner Is the Birth Mother

	Parenthood P1 (Event history)	Youngest birth mother M1 (LPM)
Marriage cohort		
1995–2002 (Ref.)		
2003–2004	1.15*** (0.18)	
2005–2008	1.59*** (0.16)	
2009–2016	1.78*** (0.15)	
Duration of marriage		
0–1 (Ref.)		
2–3 years	−0.11 (0.07)	0.09** (0.03)
>3 years	−0.77***	0.14***
	(0.10)	(0.04)
Age at marriage, younger partner		
18–30	0.19* (0.07)	−0.11*** (0.03)
31–35 (Ref.)		
>35	−1.68*** (0.11)	0.12* (0.05)
Older partner >3 years older	−0.11† (0.06)	0.11*** (0.03)
Has child before, younger partner	−0.52*** (0.12)	0.08 (0.05)
Has child before, older partner	−0.49*** (0.09)	0.11** (0.04)
Education		
No higher education (Ref.)		
Only younger partner	0.35*** (0.10)	−0.03 (0.04)
Only older partner	0.34*** (0.09)	−0.05 (0.04)
Both	0.49*** (0.08)	−0.04 (0.03)
Household income (log)	0.43*** (0.06)	
Income, younger partner (log)		0.00 (0.02)
Relative income, younger partner		0.05 (0.09)
Constant	−5.89*** (0.40)	0.47*** (0.10)
*N* couples	3,335	1,325
*N* couple years	14,445	
Pseudo *R*‐squared/*R*‐squared	0.172	0.05

*Note:* Standard errors in P1 clustered at the couple level. Robust *SEs* in parentheses. ***p < .001. **p < .01. *p < .05. †p < .1.

As expected, couples were less likely to have a child the older the younger partner was. The age difference was, for most, not decisive for their likelihood of becoming parents, although there is a negative association (significant at the 10 % level). Childbearing was positively associated with household resources; if one or both partners were highly educated, they were more likely to have a child than if neither was highly educated, and the probability that they became parents increased with household income.

The choice of birth mother in the couples who become parents is analyzed in an LPM (M1). The probability that the younger partner carried the child increased with her age (implying that the likelihood that the older partner gave birth decreased with her age). If there was a large age difference in the couple (at least 4 years), the younger partner was more likely than the older partner to be the first birth mother.

The choice of the younger partner as the birth mother was positively associated with the older partner having a child previously, but unrelated to the younger partner's previous biological parenthood, all else being equal.

Partly because the M1 analysis focuses on couples who, on average, were more highly educated than those who did not transition to parenthood (cf. P1), education was unrelated to the choice of birth mother, as was the younger partner's income and relative income the year before childbirth. Hence, the likelihood that a couple had a child was related to income and educational resources, whereas the choice of birth mother for this child was not.

### Analysis of a Second Childbirth and Birth Mother of This Child

Table 4 shows the results of the discrete‐time event history analysis of the birth of a second child (P2) and the LPM of the birth mother of the second child (M2). Overall, coefficients are as expected from the descriptive analyses and the P1 and M1 models, with one potential exception. Controlling for age at marriage, years since first birth, and so on, couples in which the first birth mother was more than 3 years younger than the social mother were less likely to have a second child compared to other couples (Table 4, P2). Sensitivity checks suggest that this relationship is driven by older couples, and the association disappears when couples are excluded in which either or both of the partners were 40 years of age or older (available from the authors on request).

**Table 4:** The Probability that a Female, Same‐Sex Parental Couple has a Second Child and that the Same Partner Is the Birth Mother of Both Children

	Second child P2 (event history)	Same birth mother M2 (LPM)
Marriage cohort		
1995–2004 (Ref.)		
2005–2008	0.82*** (0.17)	
2009–2016	0.76*** (0.18)	
Years since first child birth, five categories		
0–1 years	−2.87*** (0.18)	
2	−0.31* (0.13)	
3 (Ref.)		
4	−0.34* (0.17)	
>4 years	−1.03*** (0.18)	
Years since first child birth, three categories		
0–2 years		−0.11* (0.05)
3 (Ref.)		
>3 years		−0.01 (0.05)
Age at marriage, first birth mother		
18–30	0.36** (0.11)	−0.02 (0.05)
31–35 (Ref.)		
>35^a^	−0.70*** (0.15)	−0.14* (0.06)
Age difference		
0–3 years (Ref.)		
First birth mother >3 years younger	−0.46*** (0.12)	0.39*** (0.05)
First birth mother >3 years older^a^	0.14 (0.14)	−0.16** (0.06)
Education		
No higher education (Ref.)		
Only first birth mother	0.25† (0.15)	−0.02 (0.06)
Only first social mother	0.41** (0.15)	−0.18** (0.06)
Both	0.38** (0.13)	−0.18*** (0.05)
Household income (log)	0.09* (0.04)	
Income, first birth mother (log)^b^		0.02 (0.03)
Relative income, first birth mother^b^		−0.15 (0.14)
Constant	−2.96*** (0.49)	0.65*** (0.15)
*N* couples	1,336	510
*N* couple years	5,224	
Pseudo *R*‐squared/*R*‐squared	0.17	0.22

*Note: SEs* in P2 clustered at the couple level. Robust *SEs* in parentheses. ^a^In M2, the number of couples in this category whose second child has the same birth mother as the first child is small: 27 birth mothers were >35 at marriage and 30 were more than 3 years older than the social mother was. ^b^Measured the year before the first child's birth. ***p < .001. **p < .01. *p < .05. †*p* < .1.

The main independent variables in Table 4 are the indicators of human capital and financial resources. The log odds that a couple has a second child increased if both of the partners, or at least the social mother, was more highly educated. Worth noting is that the birth mother's higher education was also positively linked to having a second child, although significant only at the 10 % level. This may partly be due to the smaller sample in this model compared to Table 3, Model P1, suggesting that second parenthood—as well as first‐time parenthood—was less common in couples in which both were less educated. Also mirroring the results for the first childbirth, the likelihood of a second childbirth increased with household income. One contributing factor may be the costs for fertility treatment (for couples who do not use home insemination).

The final analysis in Table 4—of the likelihood that both children have the same birth mother (M2)—shows that if the first birth mother was over 35 years old (rather than 31–35) in the year of marriage, the likelihood that she was chosen as the second birth mother was lower. Holding other factors constant, this may indicate that taking turns giving birth becomes especially important when the partners are in their late thirties. As expected, the first birth mother was more likely to carry the second child if she was at least 4 years younger than her partner, and the couple was more likely to switch birth mothers if the first birth mother was at least 4 years older than the first social mother. Among those who had a second child, it was more common to switch birth mothers if both were higher educated or at least the first social mother was higher educated. Given that the same pattern is found in the P2 analysis, this suggests that second childbirth might have been more common in couples in which the first social mother was more educated, partly because she was willing to be the second birth mother and/or the couple was planning for two children—and to take turns giving birth—from the start. The second resource, income, is not related to the choice of second birth mother, neither the first birth mother's income nor her relative income. Income is measured the year before first birth.

Returning to the theoretical expectations and hypotheses, the first hypothesis received support, as couples with higher educational and financial resources were more likely to transition to parenthood. The second and third hypotheses, linked to within‐couple short‐term (specialization) and long‐term financial reasoning (relative resources/bargaining), did not receive any clear support. Within‐couple resource differences were small in the couples who transitioned to parenthood, which may explain why factors other than income and education matter for who becomes the birth mother. Nevertheless, among those with two children, taking turns giving birth was more common for couples in their upper thirties and in couples where the social mother or both parents were more educated. This may provide tentative evidence for the long‐term family utility perspective (cf. Table 1 third column, third row).

## Concluding Discussion

This article studied the transition to parenthood in female, same‐sex couples, focusing on which couples become parents and who, of the two women in couples who have a child, is chosen to be the birth mother of the first and (any) second child. Earlier research (Aldén et al., 2015; Kolk & Andersson, 2020) indicates, and our analyses confirm, that children are often born early in the marriage. In the time period in focus in this study, marriage enabled the social mother to adopt the child even if the child was not conceived at a Swedish clinic. Starting in 2019, cohabiting mothers are acknowledged as mothers from birth not only if the child is conceived at a Swedish clinic but also if conceived at a certified clinic abroad (with the use of a non‐anonymous donor). Over time, this may contribute to a decreasing association between marriage and parenthood in lesbian couples.

Based on longitudinal population register data with information on both partners in married, female same‐sex couples, our analyses show that couples are more likely to become first‐ and second‐time parents if one or both partners are highly educated compared to if neither is highly educated. This is in line with expectations and supports Hypothesis 1. The probability that couples become parents and that they have a second child is also higher in families with higher household income. Hence, even though health‐related costs for medically assisted procreation are low for a first child in Sweden, socioeconomic factors play a role as lesbian couples decide to become parents. This is in line with earlier research on different‐sex couples, indicating that many couples secure a reasonable standard of living and income before they become parents. Special for lesbian couples is the substantially increased cost of having a second child. If lesbian couples plan for two children, they may have reasons to secure a stable income already at the birth of the first. Some couples go abroad or to a private clinic to receive medical treatment and conceive a first child. Those who do need to pay the full cost of the procedure themselves and this group most likely contributes to the fairly strong, positive link between household income and the transition to first parenthood (the couple model; P1).

Among the couples who (decide to) become parents, the choice of birth mother may theoretically be linked to short‐term or long‐term family utility. The short‐term perspective predicts a within‐couple division of reproductive behavior and paid work that is in line with Becker's theory on specialization. In other words, the lowest earning partner should be the birth mother for the first and any second child (Hypothesis 2). The long‐term family utility perspective predicts that the higher earning, or more educated partner, is the one who is chosen as the first birth mother as she has the better position in the labor market and thereby can “afford” to be pregnant and take leave (Hypothesis 3). If the parents go on to have a second child, the less resourceful partner will have had time to accumulate more work experience, and the long‐term family utility perspective is strengthened if she carries the second child.

In the empirical models, higher educated couples are more likely to transition to a first birth (Model P1) but a partner's income and higher level of education is unrelated to her likelihood of bearing the couple's first child (M1). However, if a couple has a second child, the parents are more likely to switch birth mothers if the first child's social mother or both mothers are highly educated. Hence, the long‐term family utility perspective may influence the decision of who will be the birth mother in families where career‐related costs of childbirth are higher. Within‐couple income differences are small in lesbian couples. Still, opportunity costs of childbirth and leave‐taking differ by educational level, and birth mothers often take the first and the longest parental leave (Evertsson & Boye, 2018). Hence, taking turns to give birth minimizes human capital loss and the risk of negative work or career related consequences for each partner and is most likely beneficial for the couple as a whole, at least if one or both partners are higher educated. Egalitarian ideals are often stronger among the higher educated (e.g., Edlund & Öun, 2016), and this may contribute to an increased propensity to switch birth mother for a second child and to take turns using the longest leave. In sum, this provides limited evidence for Hypothesis 3 and for long‐term family utility maximization.

Overall, the results provide little support for theories of specialization and short‐term family utility maximization, according to which it would be rational to let the lowest earner invest the most in home production and childcare (e.g., Becker, 1985) (see Hypothesis 2). This does not mean that the division of work and care in same‐sex couples is unrelated to short‐term financial concerns, but it strengthens the idea that other factors are important as well. Studies on specialization and rational, financial decision‐making would benefit from taking long‐term investments in human capital and careers into account. The benefits of both partner's making such investments and minimizing human capital loss should be substantial, also in different‐sex couples. A long‐term perspective on investments in paid work and care may be particularly important in developed welfare states where high‐quality childcare is available and affordable, social security is strong, parental leaves are generous, and where it would be difficult for a family to survive on only one income.

That said, the results presented should be interpreted with the small average within‐couple income difference in mind. The utility of specialization is small in married lesbian couples transitioning to parenthood in Sweden. Still, our results suggest that couples decide to become parents when they feel that both partners have secured a reasonable income. This raises questions about the accessibility of pregnancy and childbirth for lesbian couples in Sweden. If the couple plans for two children, a secure and good income is even more important given the substantially higher cost of medically assisted procreation techniques and treatment for a second child.

Although the current study benefits from the details and quality of Swedish longitudinal population registers, there are factors that cannot be considered in quantitative analyses of couples' choices about who will be the birth mother. These factors include the value ascribed to parental roles (Leblond de Brumath & Julien, 2012) and the desire to experience pregnancy and give birth (e.g. Chabot & Ames, 2004; Hayman et al., 2015). Based on earlier research, one can still hypothesize about these potential within‐couple differences. The main advantages of this study are its generalizability (in contrast to small‐scale, nonrandom studies), that the data are not marred by reconstruction after the event, and its ability to follow the couples from the year before marriage until, if ever, they have their first and second children. Studies such as this are important for their ability to shed light on family formation and childbearing processes within an increasingly common, but rarely studied, family type. Including same‐sex couples and families in empirical, large‐scale studies is crucial for advancing theory and knowledge and for the development of family demography and sociological research today.

## Note

This manuscript has benefitted from comments and suggestions from participants at the Research Colloquium at the Department of Sociology, University of Tübingen, Germany, in November 2018; the Community, Work and Family conference in Malta, May 2019; and the conference Economics of Sexual Orientation, Linné University, Växjö, Sweden, August 2019. We are also grateful to researchers at the Swedish Institute for Social Research and the Department of Sociology, Stockholm University, in particular Linus Andersson, Cassandra Engeman, Maria Forslund, Karin Halldén, Martin Kolk, Ylva Moberg, Rense Nieuwenhuis, Dan‐Olof Rooth, and Maaike van der Vleuten for valuable feedback on earlier versions of the manuscript.

The authors gratefully acknowledge funding from the Swedish Research Council for Health, Working Life and Welfare, grant number: Forte 2014‐2347 (PI: Evertsson). This research has also received funding from the European Research Council (ERC) under the European Union's Horizon 2020 research and innovation programme awarded to Marie Evertsson (Grant Agreement No. 771770).

## Supporting information

**AppendixS1**. Supporting Information.
