# Supplementary material for: Who Gives Birth (First) in Female Same‐Sex Couples in Sweden?
Source: J Marriage Fam. 2020 Sep 12;83(4):925–41. doi: 10.1111/jomf.12727 (PMC8359361; doi:10.1111/jomf.12727)
Supplement: Supplementary file 1 — AppendixS1. Supporting Information. [file JOMF-83-925-s001.docx]

Who gives birth (first) in female same-sex couples in Sweden? Boye & Evertsson

Cell sizes for categorical variables for the sample (a) and for outcome variable=1 (b) in the regression analyses (P1, M1, P2 and M2).

|  | **P1 (Couple’s first child)** | | **M1 (First birth mother)** | | **P2 (Couple’s second child)** | | **M2 (Second birth mother)** | |
| --- | --- | --- | --- | --- | --- | --- | --- | --- |
|  | a) N couple years | b) N first births | a) N couple years | b) N younger partner birth mother | a) N couple years | b) N second births | a) N couple years | b) N same birth mother |
| *Marriage cohort* |  |  |  |  |  |  |  |  |
| 1995-2002 | 3780 | 53 |  |  |  |  |  |  |
| 2003-2004 | 1383 | 75 |  |  |  |  |  |  |
| 1995-2004 |  |  |  |  | 933 | 57 |  |  |
| 2005-2008 | 3073 | 298 |  |  | 1540 | 192 |  |  |
| 2009-2016 | 6209 | 876 |  |  | 2751 | 261 |  |  |
| *Years since marriage* |  |  |  |  |  |  |  |  |
| 0-1 year | 5812 | 800 | 808 | 413 |  |  |  |  |
| 2-3 years | 3169 | 332 | 340 | 197 |  |  |  |  |
| >3 years | 5464 | 170 | 177 | 108 |  |  |  |  |
| *Years since 1st child birth, 5 categories* |  |  |  |  |  |  |  |  |
| 0-1 years |  |  |  |  | 2498 | 48 |  |  |
| 2 |  |  |  |  | 968 | 188 |  |  |
| 3 |  |  |  |  | 644 | 153 |  |  |
| 4 |  |  |  |  | 368 | 64 |  |  |
| >4 years |  |  |  |  | 746 | 57 |  |  |
| *Years since 1st child birth, 3 categories* |  |  |  |  |  |  |  |  |
| (1) 0-2 years |  |  |  |  |  |  | 236 | 124 |
| (2) 3 years |  |  |  |  |  |  | 153 | 92 |
| (3) >3 years |  |  |  |  |  |  | 121 | 67 |
| *Age at marriage, younger/1st birth mother^a^* |  |  |  |  |  |  |  |  |
| 18-30 | 4325 | 726 | 750 | 367 | 1979 | 226 | 226 | 141 |
| 31-35 | 3533 | 429 | 428 | 250 | 2005 | 207 | 207 | 115 |
| >35 | 6587 | 147 | 147 | 101 | 1240 | 77 | 77 | 27 |
| *Cell sizes cont.* | **P1 (Couple’s first child)** | | **M1 (First birth mother)** | | **P2 (Couple’s second child)** | | **M2 (Second birth mother)** | |
|  | a) N couple years | b) N first births | a) N couple years | b) N younger partner birth mother | a) N couple years | b) N second births | a) N couple years | b) N same birth mother |
| *Age difference* |  |  |  |  |  |  |  |  |
| 0-3 years | 6887 | 704 | 718 | 350 | 2772 | 299 | 299 | 147 |
| Older partner >3 years older | 7558 | 598 | 607 | 368 |  |  |  |  |
| 1st birth mother >3 younger |  |  |  |  | 1509 | 116 | 116 | 106 |
| 1st birth mother >3 older |  |  |  |  | 943 | 95 | 95 | 30 |
| *Has child before, younger/1st birth mother^a^* |  |  |  |  |  |  |  |  |
| no | 12218 | 1217 | 1236 | 660 |  |  |  |  |
| yes | 2227 | 85 | 89 | 58 |  |  |  |  |
| *Has child before, older/first social mother^b^* |  |  |  |  |  |  |  |  |
| no | 10955 | 1141 | 1158 | 605 |  |  |  |  |
| yes | 3490 | 161 | 167 | 113 |  |  |  |  |
| *Education* |  |  |  |  |  |  |  |  |
| No higher education | 5764 | 388 | 395 | 225 | 1581 | 125 | 125 | 86 |
| Only younger/1st birth mother^a^ | 2142 | 207 | 208 | 119 | 985 | 93 | 93 | 64 |
| Only older/1st social mother^b^ | 2869 | 263 | 271 | 138 | 795 | 82 | 82 | 34 |
| Both | 3670 | 444 | 451 | 236 | 1863 | 210 | 210 | 99 |
| Total | 14445 | 1302 | 1325 | 718 | 5224 | 510 | 510 | 283 |

^a^ P1, M1: Younger partner; P2, M2: First birth mother

^b^ P1, M1; Older partner: P2, M2: First social mother
